# Tracking false-negative results in molecular diagnosis: proposal of a triplex-PCR based method for leishmaniasis diagnosis

**DOI:** 10.1186/1678-9199-20-16

**Published:** 2014-04-22

**Authors:** Suênia da Cunha Gonçalves-de-Albuquerque, Rômulo Pessoa e Silva, Rayana Carla Silva de Morais, Lays Adrianne Mendonça Trajano-Silva, Carlos Gustavo Régis-da-Silva, Sinval Pinto Brandão-Filho, Milena de Paiva-Cavalcanti

**Affiliations:** 1 Departamento de Imunologia, Centro de Pesquisas Aggeu Magalhães (CPqAM), Av. Prof. Moraes Rego, s/n, Recife, Pernambuco CEP 50670-420, Brasil

**Keywords:** Extraction control, Multiplex PCR, pUC18, Leishmaniasis, Diagnosis, False-negative result

## Abstract

**Background:**

Molecular biological methods have become increasingly relevant to the diagnosis and control of infectious diseases, such as leishmaniasis. Since various factors may affect the sensitivity of PCR assays, including DNA yield and purity, an optimal extraction method is pivotal. Losses of a parasite’s DNA during extraction may significantly impair its detection by PCR and lead to false-negative results. This study proposes a triplex PCR assay targeting the parasite’s DNA, an external control (pUC18) and an internal control (G3PD) for accurate diagnosis of leishmaniasis.

**Results:**

Two primer pairs were designed to detect the plasmid pUC18 and a triplex PCR assay targeting the *Leishmania braziliensis* kinetoplast DNA, the external control and the internal control was standardized. The triplex PCR assay was assessed for its ability to detect the three target DNA fragments simultaneously.

PCR products from pUC18 DNA resulted in bands of 368 (P1) and 316 (P2) base pairs (bp). The triplex PCR optimized with the chosen external control system (P1) allowed the simultaneous detection of the internal control (G3PD – 567 bp) as well as of small quantities (10 pg) of the target parasite’s DNA, detected by amplification of a 138 bp product.

**Conclusions:**

The new tool standardized herein enables a more reliable interpretation of PCR results, mainly by contributing to quality assurance of leishmaniasis diagnosis. Furthermore, after simple standardization steps, this protocol could be applied to the diagnosis of other infectious diseases in reference laboratories. This triplex PCR enables the assessment of small losses during the DNA extraction process, problems concerning DNA degradation (sample quality) and the detection of *L. braziliensis* kDNA.

## Background

Methods in molecular biology have become extremely relevant to the diagnosis and control of infectious diseases, such as leishmaniasis. Information on DNA sequences has been extensively exploited for the development of polymerase chain reaction (PCR) based assays for various applications, including understanding of parasite genetics and diagnosis of parasitic diseases [1]. DNA analysis offers advantages over traditional serological and parasitological methods, including decreased sample processing time and elimination of the need for culturing [2,3]. As a result, medical and veterinary diagnostic tools and public health laboratories worldwide are increasingly being called upon to introduce molecular diagnostic tests for both endemic and exotic diseases [4].

PCR and its variations (e.g., nested, multiplex, real-time) have contributed to the detection of disease agents in humans and animals, including *Leishmania* spp. with high sensitivity and specificity [5-7]. Additionally, the possibility of combining multiple targets in the same assay enables the identification of parasites to the species level, the evaluation of sample integrity and also PCR performance on pools of phlebotomine sandflies [8-12].

Recently, a duplex PCR assay was standardized to evaluate the integrity of the DNA template by amplifying the glyceraldehyde-3-phosphate dehydrogenase gene (G3PD or GAPDH) of mammals in the same reaction for the diagnosis of *Leishmania* spp. infection [7]. The quality of the DNA samples extracted from blood and biopsies was evaluated by including a primer system to detect the G3PD gene in two standardized PCR assays for *L. infantum* (mVL) or *L. braziliensis* (mACL). The expression of this gene in all mammalian cells ensures its detection in samples whose conditions are suitable for diagnostic tests [13-15]. In the aforementioned study, the endogenous control was negative in 33% of the samples tested, demonstrating losses of reliability due to poor sample quality. In addition, some known positive samples, with quality assured by the G3PD detection, were PCR-negative for the main DNA target (*L. infantum*). These results indicated the necessity of not only ensuring the high quality of each individual sample, but also assessing possible losses of minimal amounts of the target parasite’s DNA.

The extraction of nucleic acids from biological samples is a critical step and may result in losses of the target DNA [16]. Since various factors may affect the sensitivity of PCR assays, including DNA yield and purity, an optimal extraction method is essential. In recent years, commercial extraction kits have become available for blood and other biological specimens, such as skin. These kits perform direct cell lysis, speeding up sample processing and reducing the potential of variability, which has led to common use. However, extraction protocols often suffer from inadequacies including incomplete cell lysis, DNA binding to surfaces, poor DNA recovery and the co-extraction of salts and proteins that inhibit DNA-DNA hybridization and enzymatic reactions [16,17].

Realizing the importance of DNA extraction for the proper functioning of PCR-based methods, several groups have evaluated the efficiency of commercial kits for different types of samples [16,18-20]. Nevertheless, the most common PCR protocols monitor the sample quality by spectrophotometric determination of DNA or in separate reactions, neither controlling for PCR inhibition nor confirming successful DNA extraction recovery, ultimately increasing the costs and chances of false-negative results. Conversely, the detection of a known-to-be-present DNA sequence in the sample may reveal possible losses of genetic material during the purification process, thus enabling a more correct interpretation of the PCR results. Therefore, to refine the molecular diagnosis of human and canine leishmaniasis, this study assessed a molecular triplex PCR assay by targeting an external control (a commercial plasmid), an internal control (a housekeeping gene) and the target parasite’s DNA (*L. braziliensis* kinetoplast DNA).

Compared to the traditional PCR protocols, the protocol presented herein allows a better interpretation of PCR results and promote quality assurance of the leishmaniasis diagnosis. The cost-benefit ratio is improved by ensuring the quality of results sample-by-sample, along with the simultaneous detection of *Leishmania* spp. Performing three PCRs in one mixing saves reagents, which makes it a rational decision for repeating reactions. Additionally, this method may be adapted for the molecular diagnosis of any infectious disease, providing fast results with a small margin of error.

## Methods

### Blood samples and controls

Human and canine blood samples were collected from one healthy person and two healthy dogs and used as controls. These samples were used to produce known negative and positive controls for the optimization tests. Informed consent was obtained from the dogs’ owners and the person included in the study, and all procedures were approved by the Research Ethics Committee (CEP-FIOCRUZ/PE, 42/2010) and by the Ethics Committee for Animal Use (CEUA- FIOCRUZ/RJ, LW-41/10 and LW-1/11) of our institution. As positive controls, a blood sample from a healthy dog and one from the human were spiked with genomic DNA of *L. braziliensis* (MHOM/BR/1975/M2903): ~4.5 × 10^3^ parasites/mL were used, considering the detection limit (mACL = 10 pg) of the duplex PCR as determined previously [7].

### DNA extraction by commercial kit

Blood DNA extraction was performed using a commercial kit (illustra® blood genomicPrep Mini Spin Kit, GE Healthcare, USA), following the manufacturer’s instructions and comprising five basic steps: blood cell lysis, load and bind, wash 1, wash 2, and elution. After protein degradation and cell lysis, before the second step (load and bind), the plasmid pUC18 was added according to the predetermined limit of detection.

### Plasmid pUC 18, primers design and multiplex PCR standardization

The commercial plasmid pUC18 (Boehringer Mannheim, Brazil) was used to assess DNA losses during the extraction process. Two primer pairs (P1-P1*f*: 5′-GTAATAGCGAAGAGGCC-3′; P1*r*: 5′-TAAGAAACCATTATTATC-3′ and P2-P2*f*: 5′-TTGTACTGAGAGTGCAC-3′; P2*r:* 5′-CAGGAAACAGCTATGAC-3′) were designed based on two sequences available in GenBank [GenBank: L08752] and [Genbank:L09136]. PCR trials were conducted to evaluate the performance of the two pUC18 detection systems. Non-template control (NTC) and three different concentrations of pUC18 were included in each PCR run. Based on these preliminary results, the primer pair with the best performance was chosen to compose the triplex system. The detection limit of the PCR was assessed by testing ten-fold serial dilutions (from 50 ng/μL to 0.5 fg/μL) of pUC18. Concentrations between 1 and 20 μM of the forward (P*f*) and reverse (P*r*) primers were tested to determine the optimal amount of primers to be included in the duplex PCR reactions for the diagnosis of cutaneous leishmaniasis (mACL) [7].

For the first triplex PCR trials, according to the preliminary results, the plasmid pUC18 was added to negative and positive controls whereas each primer (P*f* and P*r*) was added to the mACL master mix, containing the primers previously designed [7] for internal control detection (G1*f*: 5′-ATCTTCCAGGAGCGAGATCCC-3′; G2*r*: 5′-CTGCTTCACCACCTTCTTGAT-3′). The primers kDNA*f* (5′-ATGCCTCTGGGTAGGGGCGTTC-3′) and kDNA*r* (5′-GGGAGCGCGGCCCACTATATT-3′), designated as kDNA1 system, were designed by our research group to detect the conserved region of the *L. braziliensis* kDNA. The standardization process was performed by analyzing the results of the interactions among the systems P*f/*P*r*, G3PD (endogenous control), and kDNA1. When necessary, changes were made in the cycling conditions by testing different annealing and extension temperatures performed in the Eppendorf Mastercycler gradient (Eppendorf AG, Germany). To assess the best conditions for the triplex PCR, different amounts of each reagent (dNTP, MgCl_2_ and recombinant *Taq* DNA Polymerase, Invitrogen, Brazil) were also evaluated.

### Specificity of primer pairs on triplex assays

To evaluate the specificity of the primers in a triplex PCR format, a sample containing the three targets (*L. braziliensis*, G3PD gene, and pUC18) was subjected to PCR amplification by the three primer sets (kDNA1, G3PD and P1) separately, under standardized conditions. The PCR products obtained by this experiment were purified and sequenced using an automatic sequencer (ABI Prism 3100 Genetic Analyzer, Applied Biosystems, USA). The obtained sequences were analyzed using BioEdit software (http://www.mbio.ncsu.edu/bioedit/bioedit.html) and compared with similar nucleotide sequences available in GenBank using the Basic Local Alignment Search Tool (BLASTn) (http://blast.ncbi.nlm.nih.gov).

### Gel electrophoresis and documentation

Amplicons were resolved in 1.5% agarose gel electrophoresis, stained with ethidium bromide (10 mg/mL). A 100 bp Ladder DNA (GibcoBRL Life Technologies, USA) was used as the molecular marker. Gel pictures were taken using a Kodak MI GL100 Imaging System.

## Results

**Figure 1:**
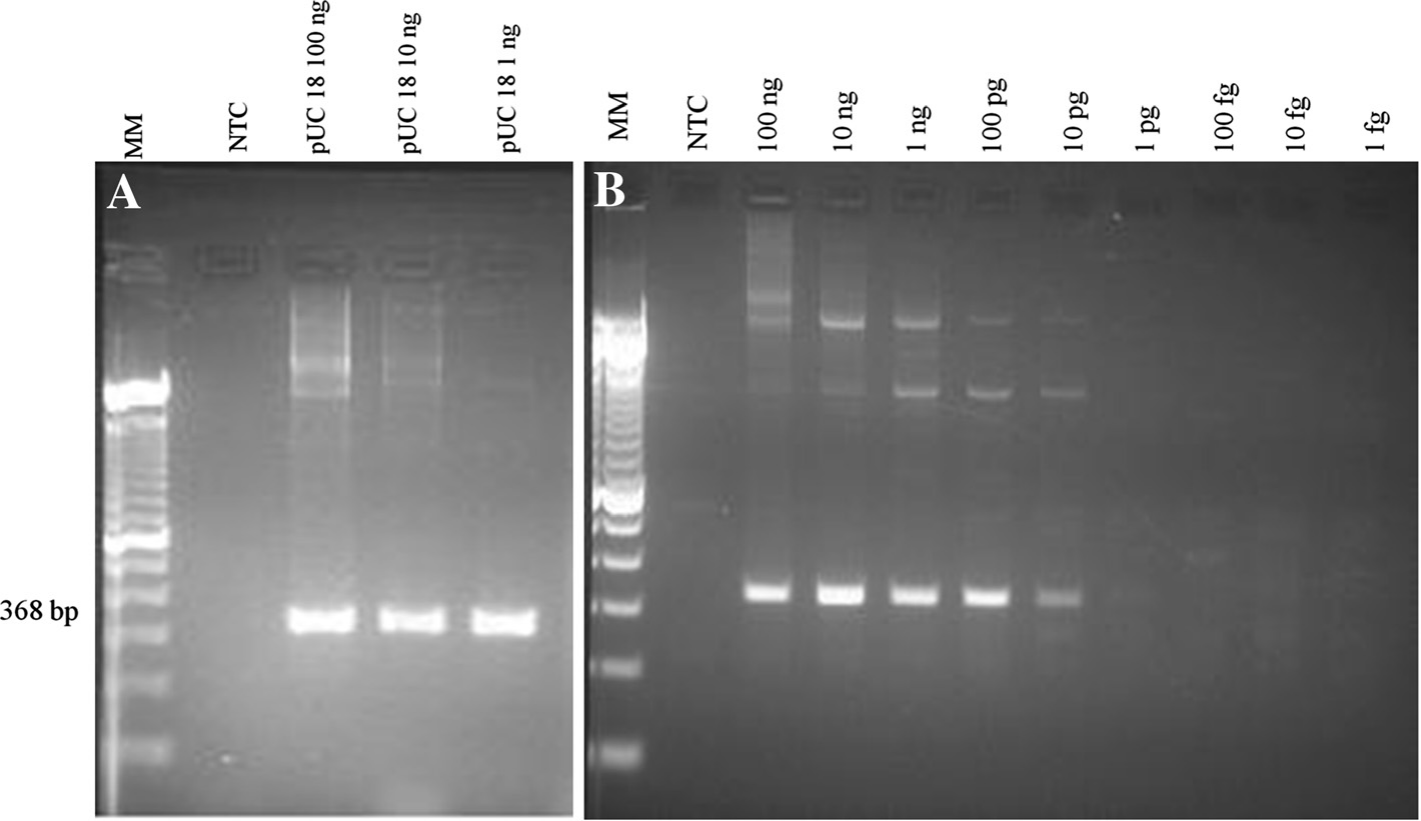
**Preliminary test of P1 primer set and detection limit determination. (A)** Preliminary test of primers P1*f*/P1*r* using different concentrations of pUC18. Note that spurious bands formed by whole plasmid electrophoresis runs can occur when using high concentrations of pUC18 in the reaction. **(B)** Sensitivity of system P1 (uniplex PCR) determined by ten-fold serial dilution (in nanograms per microliter) of pure pUC18 DNA. NTC - no template control. MM: molecular marker, 100 bp Ladder DNA (GibcoBRL Life Technologies, USA).

The sequences of the two primer pairs targeting the plasmid pUC18 were: P1*f* (5′-CGTAATAGCGAAGAGGCC-3′) and P1*r* (5′-TAAGAAACCATTATTATC-3′) for the system P1; and P2*f* (5′-TTGTACTGAGAGTGCAC-3′) and P2*r* (5′-CAGGAAACAGCTATGAC-3′) for the system P2. Amplicons from pUC18 DNA resulted in bands of 368 (P1) and 316 (P2) base pairs (bp), being suitable for multiplex PCR with G3PD (567 bp) and kDNA1 (138 bp). Based on its excellent performance (Figure 1 – A), the system P1 was chosen to compose the triplex PCR. The system P2 was not reproducible. The uniplex PCR carried out with pure pUC18 DNA was able to detect a concentration as low as 5 pg/μL (total of 10 pg per reaction mixture) as shown in Figure 1 – B. The lowest amount of primers that held this sensitivity (10 μM of each of them) was chosen to compose the first triplex PCR trials.

The observation of competition among primer pairs and target templates indicated a necessity to modify some conditions of mACL and P1 standardized initially. Simultaneous amplification of 10 pg per reaction pUC18, G3PD, and *L. braziliensis* was obtained under the following conditions: *Taq* Polymerase Buffer (0.7 μM Tris–HCl, 1.8 nM KCl, pH 8.4), 5.6 nM MgCl_2_, 40 nM dNTPs, 2 μM of each primer kDNA*f*/kDNA*r*1, 5 μM of each primer G1F/G2R, 20 μM of each primer P*f*/P*r*, 10 U of Taq polymerase and 2 μL of DNA template.

Sequences of amplicons generated with the system P1 showed high homology (100% identity) with pUC18 cloning vector [GenBank: L08752.1]. High homologies (100% identity) were also found with other cloning vectors [GenBank: JX069764.1, JQ927446.1, HQ207194.1, and FJ389180.1] and with *Bacillus subtilis* [GenBank: CP002468.1]. As to G3PD, the highest homology (75%) was found with a sequence of a human x-linked GAPD pseudogene [GenBank: X01111.1]. The kDNA1 product presented 94% similarity to a sequence of *L. braziliensis* strain MHOM/BR/75/M2904 [GenBank: FR799010.1]. Identities between 88% and 93% were also found with other species of the subgenus *Viannia* (*L. peruviana*, *L. guyanensis*, and *L. panamensis*) (data not shown).

## Discussion

The purpose of this work was to suggest a method to track possible causes of false-negative results in microbiological diagnosis by PCR, requiring minimal technical training. The triplex PCR assay proposed herein provides a rapid molecular tool to assess the occurrence of false-negative results by detecting simultaneously two quality controls and the target parasite’s DNA simultaneously.

In theory, the differences in the guanine and cytosine (GC) contents of primers used (e.g., P1 and kDNA1) could interfere in the annealing temperatures for each primer pair and eventually affect the triplex PCR performance. However, no interference was observed under the standardized conditions.

A lack of reproducibility of the P1 in detecting the extraction control was observed only in positive samples. Depending on the parasitic load in the positive specimens, the early amplification of the parasite’s DNA consumes the reagents, leading to no amplification of the DNA reporter. Indeed, the P1 system gave reproducible results during the optimization process in *L. braziliensis*-negative samples, being helpful in diagnosis interpretation and thus achieving the proposed aim. Even minimal losses of genetic material may affect significantly the detection of the parasite’s DNA, which is often found in small quantities, leading to incorrect diagnostic conclusions. As a recognized limitation, high molecular weight bands could be observed in agarosis gels, representing excess and/or folding of pUC 18 plasmid in the samples. However, the appearance of these bands does not confuse the interpretation of the results, as the amplicon size of each system is well known and was demonstrated by sequence analysis to be specific.

Against this background, this new tool provides a more reliable interpretation of the PCR results, as shown in Table 1, especially by virtue of contributing to quality assurance of leishmaniasis diagnosis. Furthermore, after simple standardization steps, this protocol could be applied to the diagnosis of other infectious diseases in reference laboratories.

**Table 1:** **Interpretation and actions suggested after observation of different positivity combinations of the quality controls and *Leishmania *** **spp. main target in the triplex PCR reaction**

**Result/PCR target positivity**	**Interpretation/meaning**	**Action**
**G3PD 567 bp (+)**	Valid results for parasite diagnosis	Conclude diagnostic test observation
**pUC 316 bp (+)**		
***L. braziliensis *** **138 bp (+)**		
**G3PD 567 bp (+)**	Valid results for parasite diagnosis despite small loss of DNA in extraction	Conclude diagnostic test observation
**pUC 316 bp (-)**		
***L. braziliensis *** **138 bp (+)**		
**G3PD 567 bp (-)**	Valid results for parasite diagnosis despite small degradation of sample	Conclude diagnostic test observation
**pUC 316 bp (+)**		
***L. braziliensis *** **138 bp (+)**		
**G3PD 567 bp (-)**	Degradation of DNA sample/presence of PCR inhibitors and/or whole DNA loss during extraction	Repeat diagnostic procedure since the blood sample collection
**pUC 316 bp (-)**		
***L. braziliensis *** **138 bp (-)**		
**G3PD 567 bp (-)**	Degradation of DNA sample before extraction	Repeat diagnostic procedure since the blood sample collection
**pUC 316 bp (+)**		
***L. braziliensis *** **138 bp (-)**		
**G3PD 567 bp (+)**	Significant DNA loss (affecting mainly parasite genome) during extraction process	Repeat the DNA extraction procedure
**pUC 316 bp (-)**		
***L. braziliensis *** **138 bp (-)**		
**G3PD 567 bp (+)**	True negative result for *Leishmania* detection assured by the positivity of quality controls	Conclude diagnostic test observation
**pUC 316 bp (+)**		
***L. braziliensis *** **138 bp (-)**		

While competition between targets was recorded initially, changes in the PCR conditions allowed us to eliminate competition problems, even while maintaining the detection limit (18 parasites per reaction), previously obtained with the mACL duplex system [7]. The simultaneous amplification of three different targets may interfere in the detection limit of PCR as compared with simplex PCR protocols, which may be able to detect minimal amounts of the parasite’s DNA [21]. The main advantage of the triplex assay presented herein is the amplification of three different targets to ensure the quality of the result. The analysis of DNA sequences showed no cross-amplification among the three primer pairs included in the triplex PCR assay. Primers designed to detect the plasmid pUC18 and the housekeeping gene G3PD did not amplify *Leishmania* spp. DNA, thereby reducing the possibilities of false-positive results to zero. Interestingly, BLASTn searches revealed that the pUC18 sequence was similar to sequences of other plasmids, which are based on the pUC18 sequence and could also be tested and optimized, according to the availability in each laboratory or research group.

The continuous refinement of PCR technologies (e.g., introduction of robotics) and the increasing demand for rapid and efficient diagnostic tools are leading to an overall reduction of costs, making PCR-based methods more accessible [22,23]. As occurs with other diagnostic methods (e.g., serology), PCR-based methods are also liable to false-positive (e.g., due to background DNA contamination) and false-negative results. False-negative results may be attributable to many factors, including low amount of template DNA in the test sample, inadequate removal of PCR inhibitors, ineffective release of microbial DNA content from the host cells, and poor DNA recovery after extraction and purification steps. As a possible solution to clarify the presence of inhibitors or degraded target DNA in samples, constitutive genes have been used in parallel assays to assess the integrity of the DNA template [7,15]. To improve the cost-benefit ratio and to shorten the time consumed by this evaluation, we recently proposed a duplex PCR assay capable of detecting a housekeeping gene as an endogenous control [7]. Nevertheless, samples from patients with low parasite burden may contain minimal amounts of a parasite’s DNA, which may be even more profoundly affected by degradation or losses during the pre-PCR stages. This fact suggests the need to include additional internal controls to monitor possible failures during critical steps, such as DNA purification. The addition of a reporter DNA template may indicate the presence of *Taq* DNA polymerase inhibitors and degraded microbial DNA, as well as losses of small DNA amounts during the extraction and purification processes.

The advantages of using internal or external controls have been demonstrated with various types of clinical samples [24-28]. For instance, an external DNA recovery standard has been developed for the determination of DNA recovery efficiency in soil samples tested by quantitative PCR [16].

In addition, the fact that the *L. braziliensis* kDNA amplicon presented homology in relation to kDNA regions of other *Viannia* species suggests that the primers and protocols reported in the study may be used directly or adapted for the detection of several species of these parasites.

## Conclusions

The triplex PCR proposed herein enables the assessment of small losses during the DNA extraction process, problems concerning DNA degradation (sample quality) and the detection of *L. braziliensis* kDNA. To the best of our knowledge, no similar method has been applied to the molecular diagnosis of parasitic diseases using blood samples. An interesting insight of our group is the comparative analysis of the single PCR to the new triplex PCR protocol to maintain high diagnosis accuracy. This method may be applied to the molecular diagnosis of any infectious disease, providing quicker results with minimal margin of error.

### Ethics committee approval

The present study was approved by the Ethics Committee for Animal Use (CEUA- FIOCRUZ/RJ, LW-41/10 and LW-1/11) of Oswaldo Cruz Foundation.

### Consent

Informed consent was obtained from the dogs’ owners and the person included in the study. Moreover, all procedures were approved by the Research Ethics Committee of Oswaldo Cruz Foundation (CEP-FIOCRUZ/PE, 42/2010).

## Competing interests

The authors declare that they have no competing interests.

## Authors’ contributions

MPC is the lead researcher of this study. MPC, SCGA and RPS designed the study. MPC and CGRS designed primers and gave intellectual support for this work. RPS, RCSM, LAMTS and SCGA performed experiments and their analysis. SCGA carried out the molecular genetic studies, analyzed amplicon sequencing and drafted the manuscript. SPBF and all authors read and approved the final version of this manuscript.
